# The Differentiation Syndrome in Patients with Acute Promyelocytic Leukemia: Experience of the Pethema Group and Review of the Literature

**DOI:** 10.4084/MJHID.2011.059

**Published:** 2011-12-04

**Authors:** Pau Montesinos, Miguel A. Sanz

**Affiliations:** Hematology Department. University Hospital La Fe, València, Spain

## Abstract

Differentiation syndrome (DS), formerly known as retinoic acid syndrome, is the main life-threatening complication of therapy with differentiating agents (all-*trans* retinoic acid [ATRA] or arsenic trioxide [ATO]) in patients with acute promyelocytic leukemia (APL). The differentiation of leukemic blasts and promyelocytes induced by ATRA and/or ATO may lead to cellular migration, endothelial activation, and release of interleukins and vascular factors responsible of tissue damage. Roughly one quarter of patients with APL undergoing induction therapy will develop the DS, characterized by unexplained fever, acute respiratory distress with interstitial pulmonary infiltrates, and/or a vascular capillary leak syndrome leading to acute renal failure. Although the development of the DS, particularly of the severe form, is still associated with a significant increase in morbidity and mortality during induction, the early administration of high-dose dexamethasone at the onset of the first symptoms seems likely to have dramatically reduced the mortality rate of this complication. In this article, we will review the clinical features, incidence, prognostic factors, management, and outcome of the DS reported in the scientific literature. We will make focus in the experience of the three consecutive Programa Español de Tratamientos en Hematología trials (PETHEMA LPA96, LPA99, and LPA2005), in which more than one thousand patients were treated with ATRA plus idarubicin for induction.

## Introduction

Differentiation syndrome (DS), formerly known as retinoic acid syndrome, is the main life-threatening complication of therapy with differentiating agents (all-*trans* retinoic acid [ATRA] or arsenic trioxide [ATO]) in patients with acute promyelocytic leukemia (APL). This complication typically occurs during induction therapy with differentiating agents while leukemic blasts are massively present, but it never take place during consolidation or maintenance therapy for APL. This syndrome is characterized by unexplained fever, acute respiratory distress with interstitial pulmonary infiltrates, and/or a vascular capillary leak syndrome leading to acute renal failure.^1^ This clinical picture may be due to cellular migration, endothelial activation, release of interleukins, and vascular factors^2^–^4^ responsible for tissue damage. Although a “cytokine storm” has been described coinciding with differentiation of blasts, the underlying etiopathogenic mechanisms of this syndrome remain partially unknown. The diagnosis of the DS is mainly based on clinical and radiological features in the context of induction therapy of APL with differentiating agents, and it usually requires the exclusion of alternative causes that could explain the signs and symptoms of the syndrome. Early therapeutic or preemptive interventions to counteract the DS using intravenous corticoids and other supportive measures should be instituted at the onset of the first suggestive signs and symptoms and before exclusion of other possible causes. The administration of high-dose dexamethasone at the onset of the first symptoms appear to reduce the DS-related mortality to 1% or less in the most recent trials.^5^–^7^

In the present article, we will review the clinical features, incidence, prognostic factors, management, and outcome of the DS. We will mainly focus on the experience of three consecutive trials of the Programa Español de Tratamientos en Hematología (PETHEMA LPA96, LPA99, and LPA2005), in which more than one thousand adult and pediatric patients were treated with ATRA plus idarubicin for induction (AIDA regimen), as well as on a review of the literature.

## Diagnosis of the DS

The first description of DS by Frankel et al,^1^ was based on the observation of a distinctive clinical syndrome in 9 out of 35 APL patients who were treated with ATRA alone for induction therapy. All of them had developed a variety of signs and symptoms characterized primarily by fever, respiratory distress, weight gain, and a chest radiograph showing pleural effusion and pulmonary infiltrates. Other clinical features, such as peripheral edema, unexplained hypotension, renal insufficiency, hyperbilirubinemia, and pericardial effusion, were also associated with the syndrome with variable frequency.

Although most subsequent studies reporting the incidence and clinical characteristics of DS in APL refer to Frankel’s definition, it must be noted that in several representative reports there is a variable interpretation of the original description. The reports of the GIMEMA-AIEOP 0493-AIDA trial,^5^ the European APL 93 trial, Errore. Il segnalibro non è definito. and the National Cancer Institute Protocol 0129,^8^ used different criteria for the diagnosis of the DS leading to a different reported incidence, ranging from 2% to 27%. The PETHEMA group has recently reported the incidence and characteristics of DS in two large series^9^,^10^ of patients undergoing induction therapy with the AIDA regimen using the same definition criteria, which were based on the presence of at least two of the following signs and symptoms: dyspnea, unexplained fever, weight gain greater than 5 kg, unexplained hypotension, acute renal failure, and, particularly, a chest radiograph demonstrating pulmonary infiltrates or pleuropericardial effusion. No single sign or symptom was considered sufficient to diagnose the syndrome, and any alternative cause explaining the clinical clinical features should be ruled out. For some authors, patients with an alternative cause explaining the syndrome have been classified as indeterminate DS, with a frequency varying from 4% to 19%,^8^,^11^ while others have used the term indeterminate DS for patients manifesting only a partial symptom complex (i.e., patients with less than 4 clinical criteria of DS).^5^ In the PETHEMA studies, a severity gradation of the DS for predicting outcomes was established, using the terms moderate and severe DS according to the number of clinical criteria fulfilled. Thus, patients with 4 or more of the above signs and symptoms were classified as having severe DS, while those with less than 4 signs or symptoms were considered to have moderate DS. In the PETHEMA series, in roughly 5% of the patients with possible DS according to the predefined criteria, an unambiguous diagnosis of DS could not be made and therefore they were not considered as having the syndrome. This was due to the presence of concurrent medical problems that could explain the clinical manifestations, such as pulmonary hemorrhage, pneumonia, congestive heart failure, renal failure, and septic shock.^9^

The diagnosis of the DS is mostly based on the above defined clinical and radiological criteria and supported by the striking response to early therapy with intravenous corticosteroids. The use of invasive diagnostic techniques, such as bronchoscopy and bronchoalveolar lavage or lung biopsy, is not usually required in patients with suspected DS and respiratory distress with lung infiltrates. Nevertheless, when the clinical course is not satisfactory despite receiving appropriate therapy, or when the patient needs orotracheal intubation for mechanical ventilation, the bronchoscopy and bronchoalveolar lavage could be useful to exclude a pulmonary haemorrhage or an infection causing the respiratory distress. Lung biopsy is usually contraindicated in patients with DS that frequently shows concomitant severe thrombocytopenia and coagulation disorders leading to an extremely high risk of bleeding complications. When performed, in life or in postmortem examination, the major hystologic findings of lung biopsies are interstitial infiltration with maturing granulocytic cells,^1^,^8^ diffuse alveolar damage, intraalveolar haemorrhage, and small vessels inflammation.^4^,^12^

## Clinical Manifestations of the DS

The typical signs and symptoms of the differentiation syndrome include fever, hypotension, dyspnea, weight gain of more than 5 kg, and pulmonary infiltrates in chest radiograph. The frequency of these signs and symptoms varies within the different reports, ranging from 59% to 95% for dyspnea, 53% to 74% for fever, 40% to 81% for pulmonary infiltrates, and 12% to 39% for hypotension. In a PETHEMA study that analyzed 739 patients included in the LPA96 and LPA96 trials, the most frequent clinical manifestations of DS were dyspnea, pulmonary infiltrates, unexplained fever, and weight gain of more than 5 kg (Table 1). Other clinical manifestations can be associated with the DS, such as peripheral edema (53% to 81%),^6^,^9^ musculoskeletal pain,^1^,^8^,^13^,^14^ acute febrile neutrophilic dermatosis (Sweet’s syndrome),^15^ and hyperbilirubinemia.^1^,^9^ However, these manifestations are generally not considered in diagnosis for DS. In addition, it is frequently observed an increased WBC count at the onset or during the development of the DS which may support the diagnosis.^8^,^11^,^14^ However, many patients will develop a full-blown DS in the absence of an increase in the WBC (e.g., during the neutropenic phase after chemotherapy or ATO). Therefore, the WBC count should not be considered as a diagnostic criterion of DS.

Abnormal findings in chest radiography or computerized tomography are very common during the course of DS (up to 81% in the severe forms).^9^ These findings will usually affect both the right and left lungs, reflecting that DS is a systemic process derived from diffuse alterations in the blood homeostasis (endothelial membrane injury by cytokines release and/or granulocyte infiltration). Treatment of DS should not be postponed until after having an abnormal chest radiograph since roughly 40 percent of patients with DS have a normal chest radiograph at presentation. The typical findings on a chest radiograph are interstitial infiltrates (i.e., septal lines and peribronchial cuffing, ground glass opacity) and pleural effusion. It is also frequent to find an increased cardiothoracic ratio (up to 87%) and parenchymal consolidation (47%) with or without air bronchogram.^12^ It should be noted that the differential diagnosis of such imaging pictures includes a wide variety of pathological conditions that could also explain the clinical picture and that should be consequently excluded to make the definitive diagnosis of DS. As an example, in patients with heart congestive failure or left ventricle insufficiency (pulmonary edema) increased cardiothoracic ratio, septal lines, and pleural effusion can be also observed in the chest radiography. An echocardiography showing ventricle dysfunction and/or prior history of cardiac disease will support the diagnosis of heart failure instead of DS. Also, parenchymal consolidation or interstitial infiltrates can be explained by pneumonia in a febrile neutropenic patient. In such cases, microbiologic isolates, patterns of fever, further response to intravenous dexamethasone or antibiotics, and the clinical and radiological course will help in making a diagnosis. Some patients developing pulmonary haemorrhage will show areas of consolidation and diffuse ground glass opacities in the chest computerized tomography or radiography that may be indistinguishable from those seen in patients with severe DS. Of note, an association between the occurrence of DS and disseminated intravascular coagulopathy and haemorragic syndrome, including pulmonary bleeding, has been reported.^9^,^15^ These finding suggests that, at least in some cases, DS and pulmonary haemorrhage may occur concomitantly as part of the same pathogenic process.

Pericardial effusion is a less frequently reported manifestation of the DS (less than 20% of cases). We should highlight that in many cases this sign is not detected in a simple chest radiography, requiring a computerized tomography or an echocardiography. In some patients the DS can be accompanied by electrocardiographic and laboratory signs of myopericarditis, with or without radiological pericardial effusion, leading to transient cardiac ventricle dysfunction.^16^ In these rare cases, the differential diagnosis between DS and acute cardiac toxicity induced by anthracyclines might be challenging. The diagnosis of DS can be supported by the fact that, in the context of differentiation therapy, myocardial dysfunction will disappear after induction phase once the DS is resolved.

## Timing of the DS

**Figure 1:**
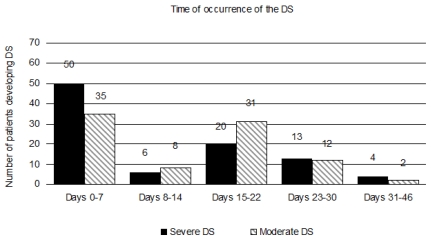


The median day of onset of clinical manifestations of the DS has been reported in a range of 7 to 12 days after starting differentiation therapy (Table 2).^17^–^19^ In a recent PETHEMA study,^9^ the DS occurred at a median of 12 days after starting ATRA treatment (range, 0–46 days). Severe DS occurred earlier (median, 6 days) than moderate DS (median, 15 days).^9^ This study showed that the presentation of the DS follows a bimodal pattern with 47% of patients developing clinical manifestations within the first week (early DS) and 48% developing symptoms during the third week or beyond after starting ATRA (late DS). A bimodal time distribution of the peak incidence of DS was observed in both moderate and severe DS (Figure 1). It should be noted that cases of early severe DS required mechanical ventilation or dialysis more frequently and were associated with a higher mortality during induction (up to 40%) compared with those occurring during the third week or beyond. Early severe DS was characterized by a higher frequency of pulmonary infiltrates and weight gain, probably related to an initial rise in leukocytosis and fluid overload. The PETHEMA study showed that the number of packed red blood cells, platelets, and fresh frozen plasma units transfused were significantly higher in patients with early severe DS compared with those with late severe DS. In contrast, hypotension, unexplained fever, pericardial effusion, and renal failure were more frequent in late severe DS. We should emphasize that in this situation distinguishing between DS and other clinical complications, particularly sepsis, may be difficult. Therefore, in these cases, treatment of DS should be complemented with therapeutic measures addressing alternative diagnoses that could also explain the symptoms (e.g., infections).

## Incidence of the DS

The reported incidence of DS in APL patients treated with standard doses of ATRA ranges from 2% to 27% (Table 2), while in patients treated with ATO ranges from 7% to 31%.^14^,^20^–^23^ This wide variation is at least partially due to the use of different diagnostic criteria employed in reported studies. In addition, the effects on the incidence and severity of the syndrome due to differences in induction therapy and preemptive and supportive measures should also be considered. The DS may also occur in patients with relapsed APL receiving salvage therapy with ATRA, ATO, or the combination of both agents.^24^ The risk of developing DS in patients with advanced disease is not well known, but it is likely that patients presenting with molecular relapse may have a lower risk of developing this complication. It should be noted that DS never develops during consolidation or maintenance therapy, since this syndrome depends on the presence of leukemic promyelocytes and the release of cytokines by these cells.

The most likely explanation for different incidence rates reported of DS in different studies could be explained by the lack of consensus in the definition, grading, and classification of the syndrome. The PETHEMA group has analyzed the incidence and severity of DS in three subsequent studies (LPA96, LPA99, and LPA2005 trials)^9^,^10^ using induction therapy with AIDA regimen. With the aforementioned criteria for the definition and gradation of DS an overall incidence of 24.8% was found in the LPA96 and LPA99 trials (12.6% severe and 12.2% moderate forms), compared with 28.5% in the LPA2005 trial (12.1% severe and 16.3% moderate). These differences in the incidence were not significant. The GIMEMA group,^5^ also using the AIDA regimen, reported a very low 2.5% incidence of DS (6 of 240 patients). However, their definition of DS was notably different to other studies. They established a diagnosis of severe DS according to the presence of 5 of the 7 signs and symptoms as originally described by Frankel et al.^1^ Interestingly, when the same definition was applied to the PETHEMA series, a similar incidence of 2.6% was found. In other studies of concurrent ATRA plus chemotherapy regimens, a greater incidence of DS has been reported (11% to 16%). Again, the diagnostic criteria for the syndrome were often not defined^25^,^26^ or were less strict compared with the GIMEMA study.

On the other hand, the concurrent use of chemotherapy with ATRA in some but not all studies has probably determined differences in the incidence and severity of DS. In this respect, the results of a randomized study that focused on patients with low WBC counts (≤ 5 × 10^9^/L),^6^ and several nonrandomized studies^25^,^26^ strongly suggested that the incidence may be lower when ATRA is administered concurrently with induction chemotherapy.

Regarding the incidence of DS in APL patients treated with ATO-based regimens for induction therapy, it seems that it may be similar to that reported in patients receiving ATRA-containing regimens. However, similarly to ATRA therapy alone, there is an apparent higher incidence and severity of the DS when induction therapy consists of ATO therapy alone.^14^ Consequently, the simultaneous administration of cytotoxic agents (i.e., hydroxyurea, cytarabine, anthracyclines) could be recommended when induction with ATO in patients presenting with hyperleukocytosis or in those developing this complication later.^20^,^23^,^24^

## Prognostic Factors for the Development of DS

Apart from a peak WBC count frequently observed at the onset of DS, very little is known about other factors predictive of DS. A study by the European APL Group analyzed prognostic factors of DS in a significant number of patients who were randomized to receive either ATRA followed by chemotherapy or both simultaneously.^6^ That study did not reveal any significant prognostic factors for DS in both treatment arms. This trial showed that patients with WBC count greater than 5 × 10^9^/L and DS tended to need mechanical ventilation more frequently than those with lower WBC counts, but this was not statistically significant and the baseline WBC count was not associated with the development of DS. In a PETHEMA study,^9^ several pretreatment variables were predictive of the severe form of this syndrome, including WBC count (> 5 × 10^9^/L), abnormal serum creatinine levels, FLT3-ITD mutation, microgranular FAB subtype, short PML-RARA isoform (BCR3), and male gender. Multivariate analysis reduced the significant variables to WBC counts and serum creatinine levels. Renal failure due to the capillary leak syndrome caused by cytokine release in DS may explain the association between high serum creatinine and severe DS. In addition, WBC count (> 10 × 10^9^/L), lactate dehydrogenase greater than the upper laboratory normal values, and peripheral blood blasts greater than 70% were significantly associated with the development of moderate DS. Nevertheless, none of these prognostic factors have been confirmed in other studies so far, and they should be cautiously interpreted. Clinical and experimental studies have suggested an association between the development of DS and the expression of adhesion molecules such as CD13^11^ and CD11b.^27^,^28^ A large PETHEMA trial9Errore. Il segnalibro non è definito. could not demonstrate such a relationship between the expression of any of the surface markers analyzed and the development of DS. On the other hand, results of a US Intergroup study indicated that, contrary to the PETHEMA study, the microgranular subtype of APL protected against the development of DS. Recently, a body mass index over 35 has been related to a higher incidence of DS,^29^ but this finding still has not been confirmed in subsequent studies.

Only one study has analyzed the factors predicting fatal outcome of DS.^30^ This study found that bad performance status (ECOG score of at least 2) and low serum level of albumin were the only prognostic factors related with DS-associated mortality. These findings may reflect the vulnerability to the end-organ effects of the DS in patients with prior poor health status.

## Management of the DS

The early addition of chemotherapy to ATRA and the administration of high-dose dexamethasone at the onset of the first sign or symptom appear to have reduced the mortality associated with this syndrome from 30%^1^,^11^ to 5% or less in the most recent trials (Table 2).^6^,^8^,^9^,^10^,^25^ It is recommended to start treatment with intravenous dexamethasone at a dose of 10 mg twice daily promptly at the very earliest suspicion of DS. Most patients will show rapid improvement and complete resolution of signs and symptoms after starting intravenous dexamethasone.^31^ Corticosteroids therapy should be continued until the complete disappearance of symptoms and then tapered. In a few cases, a first episode of DS resolved with corticosteroids can be followed by a recurring episode of DS after period without corticosteroids.^8^ In such cases, dexamethasone should be resumed until resolution of the second wave of DS. The common short-term toxic effects of dexamethasone therapy include transient diabetes, hypertension, peptic ulcer, emotional instability, and increased risk of infections. The treatment of DS with dexamethasone should include all the necessary medical support to prevent these undesirable side effects.

Whether the differentiation agents should be discontinued when DS takes place remains controversial. It seems likely that both ATRA and ATO can be safely continued in the majority of patients with DS as long as intravenous dexamethasone has been started. It is reasonable to temporarily discontinue ATRA or ATO when the DS progresses rapidly despite starting dexamethasone or when the DS is presented as a full-blown syndrome (i.e., severe DS) or with severe end-organ dysfunction (e.g., mechanical ventilation or dialysis required). Once the signs and symptoms of severe DS are completely resolved, the differentiating agent should be restarted and continued until CR and/or the achievement of minimum length duration of differentiating therapy. In this regard, the study by Thépot et al,^32^ suggested that the total duration of therapy with ATRA of less than 21 days during the induction phase was associated with higher rates of leukemic relapse. It should be noted that this study was performed in patients treated with the European APL93 and 2000 trials that did not include further administration of intermittent ATRA during consolidation phase. Temporary discontinuation of ATRA was recommended in the PETHEMA protocols in cases where there was progression of the clinical symptoms of DS or when the DS was considered life-threatening. In the first PETHEMA study,^9^ intravenous dexamethasone was administered in 90% of patients with severe DS and 82% of patients with moderate DS, while ATRA was temporarily discontinued in 64% and 60% of patients with severe DS and moderate DS, respectively. In the second PETHEMA study,^10^ intravenous dexamethasone was administered in 83% and ATRA was temporarily discontinued in 74% of patients developing DS. The overall mortality attributed to DS in both studies was 1%, suggesting that the management of the syndrome was appropriate.

Other supportive measures are also crucial in the correct management of the DS. In the PETHEMA protocols, furosemide is usually administered to treat acute renal failure, peripheral and pulmonary edema, and weight gain. Dialysis or venous continuous ultrafiltration is needed to manage some cases with refractory acute renal failure and/or fluid overload. Invasive, but also non-invasive, mechanical ventilation is indicated in some patients with severe acute respiratory failure that do not respond to high-flow oxygen therapy. In PETHEMA studies diuretics, dialysis, and mechanical ventilation were needed in 87%, 12%, and 26% of patients with DS, respectively. These supportive measures were more frequently needed in patients with severe DS than in those with moderate DS. In addition, many patients will develop pre-renal failure and hypotension in the context of a vascular leak syndrome. In those cases, careful fluids and/or vasopressor agents, in conjunction with empirical therapy with intravenous antibiotics should be implemented.

While the preemptive use of corticosteroids at the earliest clinical manifestations of DS has been adopted as the standard treatment,^33^ their prophylactic use is more controversial. The only available evidence supporting the use of corticosteroid prophylaxis is based on a study by Wiley and Firkin^34^ that was performed in 19 patients treated with ATRA alone. This study showed a reduction in pulmonary toxicity by administration of prednisone when the WBC count rose above 10 × 10^9^/L. Based on these results, several trials have included prophylaxis with dexamethasone in patients with more than 5 to 10 × 10^9^/L.^25^,^10^ The impact of prophylactic strategies for the DS was assessed in three consecutive PETHEMA trials. In the LPA96 trial, patients with a WBC count greater than 5 × 10^9^/L (before or during treatment with ATRA) received prophylaxis with dexamethasone (10 mg/12 hours intravenously for 7 days), resulting in an incidence of DS of 30%. In the LPA99 trial, all patients received DS prophylaxis with prednisone (0.5 mg/kg/d orally from days 1 to 15), and the incidence of DS was 23%. In the LPA2005 trial, only patients with WBC count greater than 5 × 10^9^/L received prophylaxis with dexamethasone (2.5 mg/m2/12h intravenously for 15 days), resulting in 28% of DS. The retrospective comparison of these trials showed an apparent reduction in the incidence of DS, but not in DS-related mortality,^30^ when a 0.5 mg/kg daily dose of prednisone during the first 15 days of induction therapy was given.

## Clinical outcome of the DS

The concomitant administration of chemotherapy and differentiating agents and the use of high-dose dexamethasone at the onset of the first symptoms appear to have reduced the DS-related mortality to 1% or less in the most recent trials, compared with 5% to 9% in the previously reported studies (Table 2). It is likely that the development of moderate forms of DS has little impact on the death rate during induction therapy, whilst the severe DS seems associated with a higher death rate. In the PETHEMA series,^9^ the DS-associated mortality was 11% in patients with severe DS, while no deaths resulted from moderate DS. Particularly, the DS-associated mortality was 16% in patients with early severe DS. Interestingly, the haemorrhage-associated mortality was also higher in patients who developed severe DS. This finding is in line with the study by Yanada et al,^35^ suggesting that patients with DS are more prone to develop severe haemorrhage during the induction phase.

The development of DS may have additional implications, such as an increase in morbidity leading to an increased use of hospital resources. In this regard, the PETHEMA group showed that the forms of severe DS leads to increased morbidity during induction therapy.9 Errore. Il segnalibro non è definito. As an example, the development of severe DS was associated with a higher incidence of thrombosis and coagulopathy, and to a higher use of plasma, platelet, and red blood cell transfusions during induction therapy. In fact, an exacerbation of the procoagulant state of APL has been concurrently observed with the development of DS,^36^–^39^ leading to an increased risk of haemorrhage and thrombosis. In addition, patients with severe DS spend more days in hospital and show grade 3 or 4 hepatotoxicity during induction more frequently.

The European APL Group has reported an increased risk of relapse independent of WBC count in patients developing DS.^7^ This interesting finding was confirmed in patients developing severe DS treated according the PETHEMA LPA96 trial, but not in those enrolled in the LPA99 trial. A possible explanation is that in the LPA96 trial most of the patients who developed severe DS received lower total dose of ATRA,^32^ since ATRA was frequently discontinued as part of the management of the DS. In contrast, patients enrolled in the LPA99 trial received additional ATRA for consolidation, which may counterbalance the reduction of total dosage of ATRA for induction therapy in patients developing severe DS. We can hypothesize that the addition of ATRA during consolidation therapy could be beneficial for reducing the risk of relapse, especially in those patients developing severe DS who had received only reduced doses of ATRA during induction. On the other hand, some authors have speculated about an increased risk of extramedullary relapse, in particular in the central nervous system, in patients developing DS.^6^,^40^ However, this association has not been confirmed in larger series.^9^,^41^

## Conclusions

Roughly one quarter of patients with APL undergoing induction therapy will develop DS. The early administration of high-dose dexamethasone at the onset of the first signs or symptoms of DS is crucial, since it appears to dramatically reduce mortality of this complication. The etiopathogenic mechanisms of this life-threatening complication are complex and remain partially unknown. Specific biological therapies to counteract the syndrome are still not available. Randomized studies are required to ascertain whether or not the use of corticosteroid prophylaxis is advantageous, particularly taking into account that infectious mortality is not apparently increased when prednisone prophylaxis is used.^30^ Risk-adapted prophylactic and therapeutic strategies based on the adverse risk factors for development of severe DS (e.g., WBC counts > 5 × 10^9^/L or abnormal levels of serum creatinine) require further research.

## Figures and Tables

**Table 1:** Signs and symptoms of moderate and severe DS during induction therapy with AIDA regimen in APL patients enrolled in the PETHEMA LPA96 and LPA9 trials (n=739).

	Frequency of the signs and symptoms according to the type of DS		
	
Clinical Signs and Symptoms	Moderate DS (n=90)	Severe DS (n=93)	
	No. (%)	No. (%)	P value
Dyspnea	53 (59)	88 (95)	<0.001
X-ray Pulmonary Infiltrates	34 (38)	75 (81)	<0.001
***Edema***	48 (53)	75 (81)	0.001
***Unexplained Fever***	48 (53)	69 (74)	0.003
Weight Gain > 5 kg	34 (38)	63 (68)	<0.001
Pleural Effusion	24 (27)	54 (58)	<0.001
Renal Failure	8 (9)	43 (46)	<0.001
Hypotension	11 (12)	36 (39)	<0.001
***Pericardial Effusion***	10 (11)	21 (23)	0.04

**Table 2:** Main publications reporting the incidence and outcome of DS in patients with APL managed with ATRA.

First author & year (reference)	Induction therapy	DS prevention	Number of patients	Indeterminate DS (n, %)	Number of DS criteria needed$	Incidence of DS n (%)	Median day of onset of the DS	Death by DS n (%)	Risk factors for DS
Frankel, 1992 (1)	ATRA	None	35	No	5 of 7	9 (26)	10	3 (9)	No
Vahdat, 1994 (11)	ATRA	None	78	15 (19)	4 of 4	21 (27)	NR	7 (9)	WBC peak, CD13+
Cortes, 1994 (18)	ATRA	CT if blasts + promyelocytes >10 ×10^9^/L	17	NR	NR	4 (24)	NR	1 (5)	No
Avvisati, 1995 (19)	AIDA	None	20	NR	2 of 3	2 (10)	10	1 (5)	NR
Mandelli, 1997 (5)	AIDA	None	240	NR	5 of 5	17 (8)	NR	1 (0.2)	NR
Wiley, 1997 (34)	ATRA	CT if WBC > 1 ×10^9^/L, PDN if WBC < 1×10^9^/L	22	NR	NR	2 (9)	NR	1 (4)	No
De Botton, 1998 (6)	ATRA followed by CT or ATRA+CT	CT if WBC >5 ×10^9^/L at presentation, if >6, >10, >15 ×10^9^/L on day 5, 10 and 15	413	NR	3 of 7	64 (15)	7	5 (1.2)	No
Tallman, 2000 (8)	ATRA	Hydrea if baseline WBC > 1 ×10^9^/L or >30 ×10^9^/L during induction	167	7 (4)	2 of 7	44 (26)	11	2 (1.2)	No
Montesinos, 2008 (9)	AIDA	DXM if WBC >5 ×10^9^/L (LPA96) or PDN 0.5 mg/kg (LPA99)	739	33 (5)	2 of 7	183 (25)	12	10 (1.4)	Creatinine > ULN, WBC >5 ×10^9^/L, LPA96
Sanz, 2009 (10)	AIDA	DXM if WBC >5 ×10^9^/L	372	9 (3)	2 of 7	106 (28)	NR	4 (1.1)	NR
Jeddi, 2009 (29)	AIDA or ATRA+CT	None or PDN 0.5 mg/kg	42	NR	3 of 7	10 (24)	NR	6 (14)	Body mass index >35*

Abbreviations: DS: differentiation syndrome, ATRA: all-transretinoic acid, AIDA: ATRA plus idarubicine, CT: chemotherapy, WBC: white blood cells, DXM: dexamethasone, PDN: prednisone, ULN: upper laboratory normal values, NR: not reported.

$ According to the following criteria: dyspnea, unexplained fever, weight gain greater than 5 kg, unexplained hypotension, acute renal failure, chest radiograph demonstrating pulmonary infiltrates, and pleuropericardial effusion.

* Predictive for all ATRA-derived complications
